# Modeling the Spread of Porcine Reproductive and Respiratory Syndrome Among Pig Farms in Lira District of Northern Uganda

**DOI:** 10.3389/fvets.2021.727895

**Published:** 2021-08-30

**Authors:** Emmanuel Hasahya, Krishna K. Thakur, Michel M. Dione, Barbara Wieland, Peter Oba, Joseph Kungu, Hu Suk Lee

**Affiliations:** ^1^International Livestock Research Institute (ILRI), Kampala, Uganda; ^2^College of Veterinary Medicine, Animal Resources and Biosecurity (COVAB), Makerere University, Kampala, Uganda; ^3^Department of Health Management, Atlantic Veterinary College, University of Prince Edward Island, Charlottetown, PE, Canada; ^4^International Livestock Research Institute (ILRI), Dakar, Senegal; ^5^International Livestock Research Institute (ILRI), Addis Ababa, Ethiopia; ^6^International Livestock Research Institute (ILRI), Hanoi, Vietnam

**Keywords:** simulation model, porcine reproductive and respiratory syndrome, Uganda, vaccination, movement control

## Abstract

Porcine Reproductive and Respiratory Syndrome (PRRS) is a viral swine disease that causes reproductive failure in breeding sows and respiratory distress in growing pigs. The main objectives were to simulate the transmission patterns of PRRS in Uganda using North American Animal Disease Spread Model (NAADSM) and to evaluate the potential effect of prevention and control options such as vaccination and movement control. The median number of infectious farms at the end of 52 weeks for the baseline scenario was 735 (36.75% of the 2,000 farms). The best effects of vaccination were observed in scenarios 60% farm coverage and 80% farm coverage, which resulted in 82 and 98.2% reduction in the median number of infectious farms at the end of the simulation, respectively. Vaccination of all medium and large farms only (33% of the farms) resulted in a 71.2% decrease in the median number of infectious farms at the end of 52 weeks. Movement control (MC) results showed that the median number of infectious farms at the end of 52 weeks decreased by 21.6, 52.3, 79.4, and 92.4% for scenarios MC 20, MC 40, MC 60, and MC 80%, respectively. This study provides new insights to the government of Uganda on how PRRS can be controlled. The large and medium farms need to be prioritized for vaccination, which would be a feasible and effective way to limit the spread of PRRS in Uganda. Scavenging pigs should be confined at all times, whether in the presence or absence of any disease outbreaks.

## Introduction

Porcine Reproductive and Respiratory Syndrome (PRRS) is a viral swine disease that causes reproductive failure in breeding sows and respiratory distress in growing pigs. PRRS virus is an enveloped positive-stranded RNA virus in the family *Arteriviridae* and order *Nidovirales* (1). In general, two distinct genotypes of the virus—Type 1 (European) and Type 2 (North American), are circulating around the world (2–4). The disease is highly contagious and spreads via direct or indirect contact (5). The main risk factors contributing to the spread of the disease include trading of pigs with unknown health status, sharing breeding boars or farm equipment, free-range rearing of pigs, unrestricted access to farms by visitors and vehicles, and swill feeding (6–8).

In Uganda, pig trade and sharing equipment and breeding boars amongst small, medium, and large pig farms have directly and indirectly contributed to numerous swine disease outbreaks (9). Pig farms are largely subsistence, with inadequate adherence to biosecurity protocols (10, 11). Moreover, they hardly invest in feeding, housing, management, and disease prevention and control, which increases the risk of disease outbreaks at the farm (11–14). The movements of pigs are uncontrolled with minimal or no enforcement of animal movement policy, resulting in the unknown health status of live animals sold into the markets (9, 15, 16). Recently, PRRS has been reported for the first time during a study to identify respiratory pathogens in Uganda (17), and a another new study detected PRRS virus from 27.4% of slaughtered pigs in Lira (18). Currently, there is no official vaccination or any other control program for PRRS in the country (13). In addition, PRRS is considered as economically important respiratory diseases and estimated to be resulting in 12–23% of production losses in Uganda (13), but the full scale of the economic impact of the disease or the cost-effectiveness of control strategies has not been studied yet.

A computer simulation model can play a key role in predicting the likely spread of a disease to help understand its magnitude, and guide policymakers about the most logical way to implement the prevention and control strategies to reduce disease transmission. Real life experiments to assess suitable control strategies would be both unethical and expensive to undertake. Therefore, this computer simulation model for PRRS in Uganda would provide useful insight into the spread of the disease and evaluate impact of different control strategies.

The North American Animal Disease Spread Model (NAADSM) is a stochastic, spatial, and state-transition disease simulation model to assess the spread of highly contagious animal diseases (19). The software provides an interface where direct and indirect contact rates and local spread parameters (derived from the field data) are used to explore the likely spread of infectious diseases and can be used to evaluate possible interventions (19). The software can be employed to study the spread of infectious diseases (20). It has been used to study the spread of PRRS and African swine fever (ASF) in Vietnam (21, 22), Canada (23), swine flu modeling between households and pig farms in Canada (24), classical swine fever (CSF), and foot-and-mouth disease (FMD) modeling in the United States (25, 26).

To our knowledge, no studies have attempted to develop the transmission of PRRS between farms that the model incorporates scavenging, sharing breeding boars and equipment–among resource-poor farms in Uganda. Therefore, the main objectives were to simulate the transmission patterns of PRRS in smallholder pig farms in Lira district of Uganda, under different types of contacts among farms using NAADSM and to evaluate the potential effect of prevention and control options such as vaccination and movement control.

## Materials and Methods

### Study Location and Population

**Figure 1:**
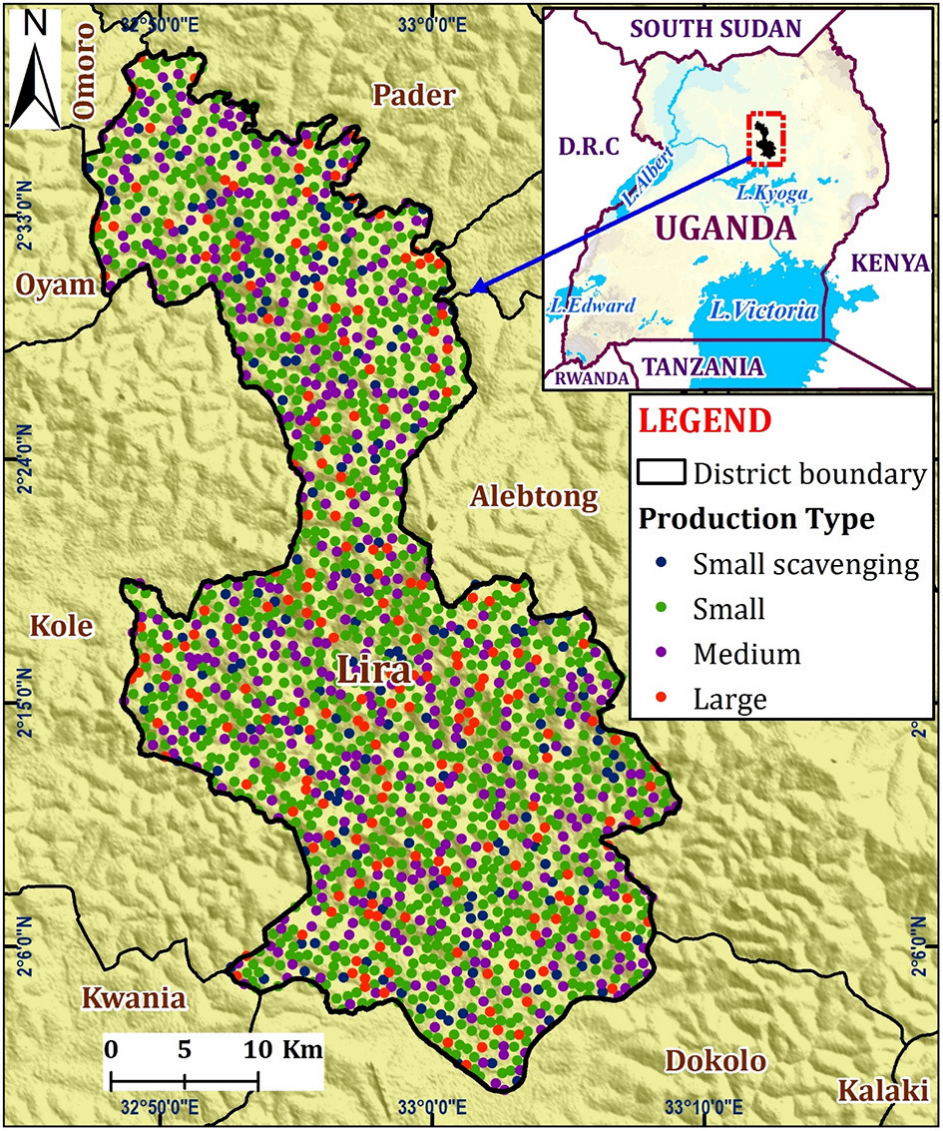
Map of Lira District showing the hypothetical distribution by the different pig farm categories.

According to the national animal census of 2009, 9.3% of all households in Lira district, Uganda are involved in pig farming (27). To develop the PRRS transmission model, the estimated number of pig farms in Lira district (total = 2,000) was obtained from the District Veterinary Office. However, the exact farm locations were not available. Therefore, the farm locations were randomly generated within the district, with the minimum distance (between farms) being 0.1 km using QGIS (Quantum GIS development 2021, QGIS version 3.14.1) (Figure 1).

The pig farms were classified into four production types based on their farm size: small-scale confined (1–4 grown pigs), scavenging small scale (1–4 grown pigs), medium scale (5–11 grown pigs), and large scale (12–30 grown pigs). The distribution by production type was 58.5% for small, 8.5% for small scavenging, 23% for medium, and 10% large-scale farms (Table 1) (11). The scavenging farms let pigs roam around during the day or night or both as they scavenge for their feed (31).

**Table 1:** Definition of parameters and values used for simulation of the spread of PRRS virus among pig farms in Lira district, Uganda, and their source references.

**Parameter**	**Value**	**References**
Total number of farms	2,000	Lira district veterinary office
Small	1,170 (58.5%)	(11)
Small scavenging	170 (8.5%)	(11)
Medium	460 (23%)	(11)
Large	200 (10%)	(11)
Infectious clinical period (small scale and small scavenging)	BETAPert (6, 8, 12 weeks)	(28)
Infectious clinical period (Medium)	BETAPert (8, 10, 16 weeks)	(28)
Infectious clinical period (Large)	BETAPert (8, 12, 22 weeks)	(28)
Natural immunity	26.5 weeks	(29)
**Transmission probabilities**		
Probability of transmission by direct contact	1	(21, 23, 30)
Probability of transmission by indirect contact	0.1	(21, 23, 30)
Distance for only direct contact	BETAPert distribution (0.1, 5, 20)	The shortest distance between farms (0.1 km), the most likely distance: (5 km); maximum distance: 20 km because farmers purchase from within their sub-county of which, on average, sub-counties are 20km in diameter
Distance for indirect contact (by scavenging and equipment sharing)	BETAPert distribution (0.1, 1, 3)	(31)

### Model Development and Parameters Estimation

The NAADSM was used to develop a stochastic simulation for the spread of PRRS virus among pig farms in Lira district. The simulation model required three key parameters: (1) probability of transmission per contact type; (2) distance between farms; and (3) mean weekly contact rates between the farm categories (Table 2). The model selected the number of contacts that arose from each infectious farm by sampling from a Poisson distribution whose mean was the production types' contact rate per simulation week. The distance was stochastically selected from a movement distance distribution for each contact from an infected farm. Then, the model chose the recipient farm from all eligible recipient farms whose distance from the source was within the assigned distance distribution (19).

**Table 2:** Weekly direct and indirect contact rates among pig farms of four production types in Lira district, Uganda used for the simulation of PRRS virus spread.

**Farm combination**	**Weekly direct contact by pig purchase**	**Weekly direct contact by boar sharing**	**Weekly direct contact (pig purchase and boar sharing)**	**Weekly indirect contact rates by equipment sharing only**	**Weekly indirect contact by scavenging alone**	**Weekly indirect contact rates (scavenging and equipment sharing)**
Small to small	0.0192	0.0308	0.0501	0.6833	–	–
Small to scavenging	0.0192	0.0308	0.0501	0.6833	–	–
Small to medium	0.0192	0.022	0.0412	0.5	–	–
Small to large	0	0.024	0.024	0.3	–	–
Scavenging to small	0.0192	0.0308	0.0501	0.6833	–	–
Scavenging to scavenging	0.0192	0.0308	0.0501	0.6833	7	7.6833
Scavenging to medium	0.0192	0.022	0.0412	0.5	–	–
Scavenging to large	0	0.024	0.0240	0.3	–	–
Medium to small	0.0192	0.0247	0.0440	0.25	–	–
Medium to scavenging	0.0192	0.0247	0.0440	0.25	–	–
Medium to medium	0.0192	0.022	0.0412	0.5	–	–
Medium to large	0	0.0192	0.0192	0.25	–	–
Large to small	0.0192	0.0538	0.0731	3.75	–	–
Large to scavenging	0.0192	0.0538	0.0731	3.75	–	–
Large to medium	0.0192	0.0481	0.0673	0.25	–	–
Large to large	0.0192	0.0192	0.0385	0	–	–

To estimate the model parameters, 80 pig farmers in Lira district were interviewed to establish contact patterns with other farms. This information was used to estimate the direct/indirect contact rates between farms by production type. The direct contact between farms comprised introducing new pigs and boar sharing for breeding purposes, while the indirect contact referred to equipment movement between farms and scavenging under the free-range production system. To obtain information on direct contacts, questions were asked on how many times pigs were brought into the farm from somewhere else and how many times a boar was shared in the previous 12 months. For indirect contact, the farmers were asked how many times they shared equipment between farms and how often they let their pigs scavenge in the previous month. All mean direct and indirect contact rates between surveyed farm types were calculated on a weekly basis given the virus can be infective for a week in the environment (32).

Direct contact distance was modeled using a BetaPERT distribution where the following values were used: minimum distance = 0.1 km, most likely value = 5 km, and maximum = 20 km. The distance was based on the fact that pig farmers share boars or purchase pigs within the sub-county they reside in, which is on average 20 km in diameter. For indirect contact, we used 0.1 km as the minimum distance between farms, 1 km as the most likely value, and 3 km as the maximum value. This distance was based on a study on the distance covered by scavenging pigs in the western region of Kenya bordering Uganda, assuming similar farming conditions to the study area (31). The transmission probabilities of PRRS virus were considered as 1 for direct contact and 0.1 for indirect contact (23, 33).

### Simulation Model Structure and Outcome

A susceptible-infectious-recovered (SIR) transmission model was used to simulate the spread of the PRRS virus among the farms, representing different production types. In addition, a continuous flow system was assumed as it is widespread in Lira district, where new pigs are introduced before the current pigs of the farm are completely removed, thereby allowing inter-mixing of animals of different origin and extending the infection duration. The model assumed that a constant herd size was maintained and did not import infected pigs from other districts during the study period. In addition, it was assumed that all farms were free of PRRS at the start of all simulation scenarios and that none of the pig herds had immunity to the virus since vaccination is not practiced in Lira district. The infectious period for different production types was extrapolated from the individual animal's infectious period (28). After the introduction of infection, large farms remained infectious for minimum = 8 weeks, most likely period = 12 weeks and maximum = 22 weeks; medium farms remained infectious for minimum = 8 weeks, most likely period = 10 weeks, and maximum = 16 weeks; and small confined and small scavenging farms remained infectious for a minimum period of 6 weeks, the most likely period of 8 weeks, and a maximum of 12 weeks. After recovery from infection, all farms remained immune for 26.5 weeks (29). The model was simulated for 52 weeks with 1,000 iterations. Each farm had the same chance to contact other farms, given the distance between pre-defined farm production types.

A total of 17 experimental model scenarios were developed and evaluated. Scenarios SC1–SC6 were intervention-free spread scenarios, with SC1 = direct and indirect contact spread, SC2 = direct contact only, SC3 = indirect contact only, SC4 = indirect spread by equipment sharing only, SC5 = indirect spread by scavenging only (scavenging and fomites), and SC6 was direct contact and equipment sharing only without scavenging. A small confined pig farm was infected for scenarios SC1, SC2, SC3, SC4, and SC6; while for SC5, a small scavenging farm was the index infected farm. We evaluated the effectiveness of different interventions, including vaccination strategies and movement restrictions among farms. The vaccine was assumed to be a modified live vaccine (MLV) and considered to be 80% effective (34, 35). The farms were vaccinated before the outbreak and had long-term immunity for the entire simulation. Various farm-level immunization coverages were applied with 20, 40, 60, or 80% of all the farms selected (VC1–VC4 scenarios). In addition, three more vaccination scenarios were considered: (1) all the medium-sized farms only (23% of the 2,000 farms, VC5 scenario); (2) all the large farms only (10% of the 2,000 farms, VC6 scenario); and (3) all medium and large farms (33% of the 2,000 farms, VC7 scenario). This study also tested the effectiveness of movement control by 20, 40, 60, and 80% reduction in contact for scenarios (MC1, MC2, MC3, and MC4), respectively, as a comparison to the baseline (SC1). The movement restrictions were assumed to be implemented 4 weeks after the initial outbreak started and maintained until the end of each simulation. It was reasoned that 4 weeks into the outbreak, the disease cannot spread widely; therefore, movement control could curtail the spread of swine diseases (36).

Finally, we conducted sensitivity analysis to evaluate the impact of direct contact transmission probability, which was changed from 1 (in the baseline scenario) to 0.75, 0.5, and 0.25. In addition, the indirect contact transmission probability was altered to 0.025 and 0.2, from 0.1, and all scenarios were compared to the baseline. Sensitivity analyses for vaccine efficacy and movement control were also carried out. It was evaluated by a reduction in vaccine coverage (from 80 to 20%) and vaccine efficacy (from 80 to 50%). The timing for the introduction of movement control was adjusted from 4 weeks to 6 and 8 weeks for each of the 20, 40, 60, and 80% reduction in movement.

We have summarized and presented the following two main model outputs: (1) the median number of infected farms for the entire simulation was the median number of all farms (cumulative) infected with PRRS virus during the 52 week simulation period; and (2) the median number of infectious farms at the end of 52 weeks was the median number of farms that were infectious at the end of the simulation (week 52), representing a snapshot of infectious farms at a given time point of 52 weeks.

## Results

Eighty farmers (50 female and 30 male) were interviewed in Lira district, representing 55 small confined farms, eight small scavenging farms, 11 medium farms, and six large farms. The respondents' mean age was 39 years, with an age range of 18–76 years old. The median number of infectious farms at the end of 52 weeks for the baseline scenario (SC1) was 735 (36.75% of the 2,000 farms) (Table 3). When only direct contact spread (scenario SC2) was modeled, a total of 106 farms (5.3%) were infectious at the end of 52 weeks. In contrast, indirect contact only (scenario SC3) resulted in 39 infectious farms (1.95%) at the end of 52 weeks. Scenario SC4 (spread by equipment sharing alone) had 20 farms infectious at the end of 52 weeks (1%), while scenario SC5 (spread by scavenging only) had five infectious farms (0.25%) at the end of 52 weeks. Thirty-five percent of the farms were infectious at week 52 when PRRS virus spread by scavenging was not included in scenario SC6.

**Table 3:** Summary of PRRS virus spread simulation among pig farms in Lira district, Uganda for baseline, direct only, and Indirect only scenarios.

	**Scenario**	**Median number of infectious farms at the end of 52 weeks (5th, 95th percentile)**	**Proportion of infectious farms at the end of 52 weeks**	**Peak Week**	**Median number of infected farms for the entire simulation (5th, 95th percentile)**				
					**Overall**	**Small**	**Small scavenging**	**Medium**	**Large**
SC1	Baseline	735 (550, 845)	36.75%	52	1,800 (1,407, 1,842)	995 (734, 1,023)	178 (162, 190)	434 (347, 444)	192 (153, 197)
SC2	Direct only	106 (20, 203)	5.3%	52	79 (1, 460)	26 (1, 166)	22 (0, 102)	22 (0, 130)	11 (0, 66)
SC3	Indirect	39 (7, 81)	1.95%	52	92 (1, 229)	43 (1, 112)	19 (0, 44)	20 (0, 49)	10 (0, 26)
SC4	Indirect spread by equipment sharing only	20 (3, 46)	1%	52	51 (1, 134)	25 (1, 65)	9 (0, 21)	12 (0, 30)	5 (0, 16)
SC5	Indirect spread by scavenging only	5 (3, 8)	0.25%	52	9 (4, 10)	NA	9 (4, 10)	NA	NA
SC6	Direct and indirect contact without scavenging	703 (510, 823)	35.2%	52	1,782 (2, 1,828)	986 (1, 1,019)	174 (1, 180)	432 (0, 443)	192 (0, 197)

**Figure 2:**
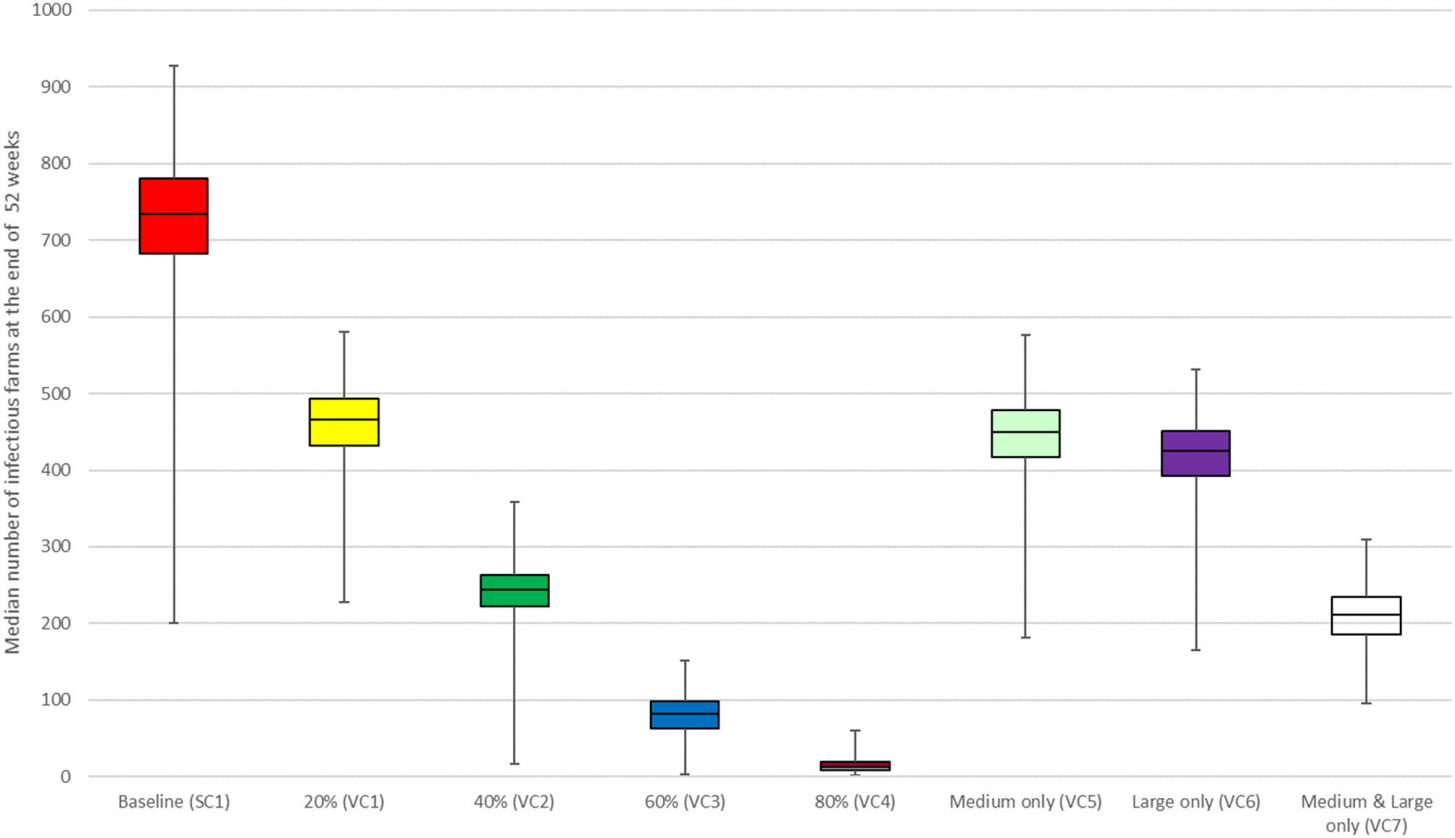
Results of vaccination coverage scenarios compared with the baseline scenario for the between-farm spread of PRRS virus in Lira District, Uganda. Individual box plot represents the median number of infectious farms from 1,000 iterations at the end of week 52. The top, middle line, and bottom of the box represent the 25th, 50th and 75th percentiles, respectively, and the end of the whiskers represent maximum and minimum.

**Figure 3:**
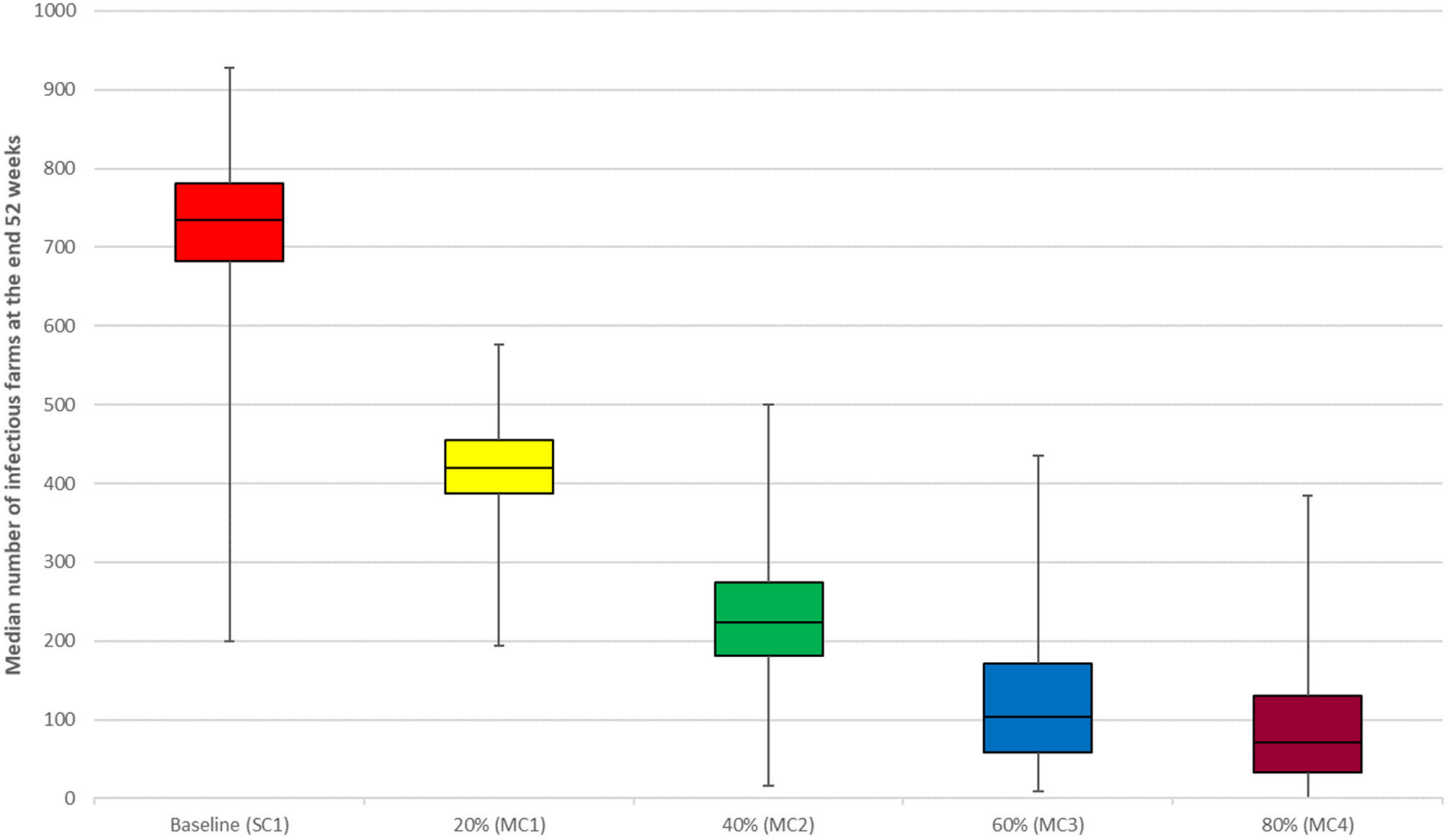
Results of movement control scenarios compared with the baseline scenario for the between-farm spread of PRRS virus in Lira District, Uganda. Individual box plot represents the median number of infectious farms from 1,000 iterations at the end of week 52. The top, middle line, and bottom of the box represent the 25th, 50th and 75th percentiles, respectively, and the end of the whiskers represent maximum and minimum.

We observed a reduction in the median number of infectious farms at the end of 52 weeks with increasing vaccination coverage (VC1, VC2, VC3, and VC4) (Figure 2). The best effects of vaccination were observed in scenarios VC3 and VC4, which resulted in an 82 and 98.2% reduction in the median number of infectious farms at the end of the simulation, respectively. Vaccination of all medium and large farms only (scenario VC7) resulted in a 71.2% decrease in the median number of infectious farms at the end of 52 weeks, compared to a reduction of 38.8 and 42.2% observed for scenario VC5 and VC6, respectively. Movement control results followed a similar trend as the vaccination intervention. The median number of infectious farms at the end of 52 weeks decreased by 21.60, 52.3, 79.4, and 92.4% for scenarios MC1, MC2, MC3, and MC4, respectively (Figure 3).

For sensitivity analysis, reducing the direct contact transmission probability from 1.0 to 0.75, 0.5, and 0.25 resulted in a decrease in the median number of infectious farms by 15.8, 34.6, 57.8% at the end of 52 weeks, respectively (Table 4). The change in indirect contact transmission probability from 0.1 to 0.05 and 0.2 led to a change in the median infectious farms by −29.8 and −36.5%, respectively. We also conducted a sensitivity analysis for the PRRS vaccine efficacy of 70, 60, and 50%, resulting in a slight general reduction in the percentage of infectious farms at the end of week 52 with decreasing vaccine efficacy (Supplementary Table 1). Sensitivity analysis for movement control when the timing for the movement restriction was altered from 4 to 6 and 8 weeks resulted in a slight change in the percentage median epidemic size from the baseline movement control scenario (SC1) (Supplementary Table 2).

**Table 4:** Summary of sensitivity analysis on the direct and indirect contact transmission probabilities.

**Scenario**	**Direct contact transmission probability**	**Indirect contact transmission probability**	**% change in direct contact transmission probability**	**% change in indirect contact transmission probability**	**Median infected farms for the entire simulation (5th, 95th percentile)**	**Median number of infectious farms at the end of 52 weeks (5th, 95th percentile)**	**% change in infectious farms at the end of 52 weeks as compared to the baseline**
Baseline	1	0.1	–	–	1,800 (1,407, 1,842)	735 (550, 845)	–
SA1	0.25	0.1	−75	–	754 (1, 1,125)	310 (177, 401)	−57.8%
SA2	0.5	0.1	−50	–	1,418 (1, 1,597)	481 (334, 575)	−34.6%
SA3	0.75	0.1	−25	–	1,689 (1, 1,759)	619 (458, 723)	−15.8%
SA4	1	0.05	–	−50	1,446 (1, 1,579)	516 (347, 618)	−29.8%
SA5	1	0.2	–	100	1,946 (1,929, 1,967)	1,003 (837, 1,124)	36.5%

## Discussion

This study was the first between-farm simulation model of PRRS virus spread among pig farms in Uganda and evaluated the effect of disease control options such as vaccination and movement restriction. Our model resulted in 36.75% of the total number of farms infectious at the end of the simulation, which is not very different from the finding of a recent survey that reported 29.8% farm-level prevalence (95% confidence interval:19.4–41.7) in Lira district (unpublished data). The model included the spread of PRRS virus via scavenging, sharing breeding boars, and sharing equipment which are very common practices in East African pig farms, including Uganda (10, 37). Scavenging was not considered in PRRS simulation models in other countries, making this model unique (21, 23, 38). Other studies suggested that boar sharing was a critical contributor to the spread of swine diseases where other forms of direct contact (pig trade) have been halted, which was observed in our study (11, 14).

Prior studies suggested that the practice of equipment sharing was associated with the spread of infectious diseases, which was consistent with our findings (39, 40). The high indirect contact rates between large and small farms in Uganda could be partly because large farm owners can afford farm equipment as opposed to the surrounding small farm owners who frequently borrow the equipment (11, 17). In contrast, large farms are spatially far away from each other, explaining why no indirect contact through equipment sharing between them was recorded from the field survey. The true reflection of the reality of this model was that 67% of the modeled farms (small confined farms and scavenging farms) were close to each other in the simulation as these farms are geographically clustered in some communities in the real world and are known for poor biosecurity (8, 41).

As expected, an increase in vaccination coverage resulted in a greater reduction of infectious herds at the end of 52 weeks, with the best results observed at 60 and 80% vaccine coverage. This trend was observed in other studies in Vietnam (21) and the United States of America (38). Due to the high contact rates from large farms to other farms, vaccination of large and medium farms resulted in a high reduction of infectious herds at the end of 52 weeks. Therefore, vaccinations need to be prioritized for the large and medium farms not only because vaccines are more likely to be afforded by these farmers than small farm owners, but also because PRRS can persist longer, after an outbreak in the larger and medium farms due to their larger size (42). Additionally, large and medium farms have a high frequency of contact (both direct and indirect) with the small farms which in case of an outbreak in such a farm would lead to farther spread to many farms; and lastly, the economic losses as a result of a PRRS outbreak on large and/or medium farms would be enormous for the owners (10, 21).

However, subsidizing vaccine prices via vaccination campaigns by the government through partnerships with development agencies could prove essential to helping small-scale farmers adopt the vaccination intervention (43). Nevertheless, studying willingness to vaccinate among pig farmers and identifying possible vaccination barriers is equally recommended as resistance to vaccination campaigns is not uncommon in Uganda (44).

Even though pig movement restrictions during disease outbreaks have received resistance in the past from pig traders, movement control after an outbreak has been proven to effectively control the spread of animal diseases (45, 46). In this study, movement restriction on direct and indirect contact also proved essential in controlling the outbreak. Even a 40% reduction in movement effectively reduced the median number of infectious farms at the end of 52 weeks by 70%. However, the movement restriction modeled herein on direct and indirect contact may not be realistic in the Ugandan context. In Uganda, control is likely to concentrate around only animals and animal products and not indirect contact forms such as equipment sharing, pig roaming (scavenging), personnel, and vehicle movement between farms (45, 46). One of the main reasons could be due to some socio-cultural factors in rural areas of Uganda that affect the effectiveness of movement restrictions (45). Therefore, proper awareness and training are necessary to improve biosecurity practices at the farm level (46).

Messaging efforts to sensitize farmers to reduce indirect contact should include ceasing the sharing of equipment, confining scavenging pigs, restricting access to farm premises, and ensuring that traders follow biosecurity protocols while accessing farms. Veterinarians and animal health professionals should comply with suitable biosecurity protocols, including and not limited to disinfection of their vehicles and their outfits at the farm. These actions are crucial in upholding biosecurity and preventing disease introduction into farms (17, 47, 48).

The main limitation of our study was that the model validation was not available as a comparison to the real situations, because there was no systematic surveillance information across the country. Furthermore, the probabilities of transmission used in this model were derived from other studies not in Uganda or in sub-Saharan Africa, which may not be representative of PRRS spread in Lira district (23, 33). It could have been possible that the transmission probability of direct contact “1” was overestimated given the conditions present in Ugandan farms. However, a sensitivity analysis was conducted to evaluate the effect of the direct probability. It has been suggested that direct contact transmission probability used in the simulation did not have an enormous impact on the model outcomes; a 50% reduction in the parameter individually resulted in <35% reduction in the median number of infectious farms at the end of 52 weeks. Our study did not establish the compliance levels of farmers to adherence to movement restriction of both direct and indirect contact as a strategy to curtail transmission of PRRS. Future studies are necessary to evaluate not only compliance to movement restrictions but also the ability to adopt vaccination against PRRS.

To conclude, this study provides new insights to the government of Uganda on how PRRS can be controlled. Vaccinating 80% of all farms would be ideal for halting the spread of the virus. However, if vaccination is widely adopted by the large and medium farms, it would prove to be a promising strategy in preventing and controlling the disease given their high frequency in direct and indirect contact with small farms than it is *vice-versa*. It is also worth noting that the bigger the herd the more likely it is for PRRS to remain persistent in such a farm. Scavenging pigs should be confined at all times, whether in the presence or absence of any disease outbreaks. Early detection and response for movement restriction are very important for implementing control strategies which require increased farmer awareness and improved overall surveillance by veterinarians and other animal health workers.

## Data Availability Statement

The original contributions presented in the study are included in the article/Supplementary Material, further inquiries can be directed to the corresponding author/s.

## Ethics Statement

This study was approved by the Institutional Review Board (IRB) of School of Biotechnical and Laboratory Sciences (SBLS), College of Veterinary medicine, Animal resources and Biosecurity (COVAB), and Makerere University. The Uganda National Council of Science and Technology (UNCST) also approved the study.

## Author's Note

Porcine reproductive and respiratory syndrome is a viral disease of pigs which causes infertility in sows and respiratory disease in young pigs. The disease is slowly spreading throughout Ugandan pig farms leading to economic losses to pig farmers. In Uganda, this disease has not been modeled to understand its dynamics in the country which hinders its control and prevention. We therefore used a software called North American Animal Disease Spread Model (NAADSM) to simulate the transmission of the disease and to evaluate vaccination and movement restrictions as control strategies. The results from this study show that vaccination of farms before the disease enters them is key to preventing the disease. Other effective control measures are restriction of movement of pigs between farms especially through trade, boar sharing, and complete stoppage of allowing pigs to roam and scavenge. This study shall help guide policy makers and implementors about the best control strategies to curtail the spread of not only this disease but also other infectious pig diseases among pig farms in Uganda.

## Author Contributions

EH, KT, MD, and HL designed the research. EH, PO, and HL executed the research. EH and HL analyzed data. EH wrote the first draft of the manuscript. KT, MD, BW, JK, and HL reviewed the manuscript. All authors contributed to the article and approved the submitted version.

## Conflict of Interest

The authors declare that the research was conducted in the absence of any commercial or financial relationships that could be construed as a potential conflict of interest.

## Publisher's Note

All claims expressed in this article are solely those of the authors and do not necessarily represent those of their affiliated organizations, or those of the publisher, the editors and the reviewers. Any product that may be evaluated in this article, or claim that may be made by its manufacturer, is not guaranteed or endorsed by the publisher.
