# Supplementary material for: Modeling the Spread of Porcine Reproductive and Respiratory Syndrome Among Pig Farms in Lira District of Northern Uganda
Source: Front Vet Sci. 2021 Aug 30;8:727895. doi: 10.3389/fvets.2021.727895 (PMC8435599; doi:10.3389/fvets.2021.727895)
Supplement: Supplementary file 1 [file Table_1.DOCX]

Supplementary Table 1. Summary of sensitivity analysis on vaccine coverage & efficacy on the median number of infected farms at the end of 52 weeks

| **Scenario** | **Vaccine**  **coverage** | **Vaccine**  **Efficacy** | **Median number of infected farms for the entire simulation**  **(5^th^, 95^th^ percentile)** | **Median number of infectious farms at the end of 52 weeks**  **(5^th^ ,95^th^ percentile)** | **% change in infectious farms at the end of 52 weeks as compared to baseline** |
| --- | --- | --- | --- | --- | --- |
| **Baseline** | ____ | ____ | **1,800 (1407, 1842)** | **735** (550,845) | ____ |
| **VC1** | **20%** | **80%** | **1,326 (1,1458)** | **466 (356,528)** | **-36.6%** |
| SV1 | 20% | 70% | 1,395 (1,1530) | 503 (354,570) | -31.6% |
| SV2 | 20% | 60% | 1,461 (1,1583) | 522 (395,591) | - 29% |
| SV3 | 20% | 50% | 1,504 (1,1641) | 554 (404,621) | -24.6% |
| **VC2** | **40%** | **80%** | **737 (1,905)** | **244 (185,292)** | **-66.8%** |
| SV4 | 40% | 70% | 896 (1,1026) | 289 (215,344) | -60.7% |
| SV5 | 40% | 60% | 1,023 (1,1175) | 345 (241,408) | -53% |
| SV6 | 40% | 50% | 1,172 (1,1315) | 402 (294,463) | -45.3% |
| **VC3** | **60%** | **80%** | **199 (1,364)** | **82 (22,120)** | **-88.8%** |
| SV7 | 60% | 70% | 347 (1,533) | 136 (78,179) | -81.5% |
| SV8 | 60% | 60% | 512 (1,741) | 189 (119,239) | -74.3% |
| SV9 | 60% | 50% | 788 (1,973) | 269 (191,322) | -63.4% |
| **VC4** | **80%** | **80%** | **30 (1,105)** | **13 (2,33)** | **-98.2%** |
| SV10 | 80% | 70% | 78 (1,210) | 34 (8,68) | -95.4% |
| SV11 | 80% | 60% | 155 (1,357) | 78 (26,112) | -89.4% |
| SV12 | 80% | 50% | 389 (1, 611) | 159 (98,202) | -78.4% |

Supplementary Table 2. Summary of sensitivity analysis on the effect of the time taken to institute movement restrictions after the outbreak on the median number of infected farms at the end of 52 weeks.

| **Scenario** | **Movement control percentage** | **Time after the outbreak** | **Median number of infected farms for the entire simulation**  **(5^th^, 95^th^ percentile)** | **Median number of infectious farms at the end of 52 weeks**  **(5^th^, 95^th^ percentile)** | **% change in infectious farms at the end of 52 weeks as compared to the baseline** |
| --- | --- | --- | --- | --- | --- |
| **Baseline** | ____ | ____ | **1,800 (1407,1842)** | **735** (550,845) | ____ |
| MC1 | 20% | 4 weeks | 1,411 (261,1548) | 420 (311,502) | -42.8% |
| SM1 | 20% | 6 weeks | 1,414 (675,1558) | 418 (318,522) | -43.1% |
| SM2 | 20% | 8 weeks | 1,422 (865,1573) | 424 (299,536) | -42.3% |
| MC2 | 40% | 4 weeks | 858 (1, 1135) | 223 (125,348) | -69.7% |
| SM3 | 40% | 6 weeks | 880 (469,1160) | 228 (140,426) | -69% |
| SM4 | 40% | 8 weeks | 930 (2,1164) | 244 (140,395) | -66.8% |
| MC3 | 40% | 4 weeks | 371 (15,701) | 103 (23,276) | -86% |
| SM5 | 60% | 6 weeks | 387 (108,741) | 112 (30,309) | -84.8% |
| SM6 | 60% | 8 weeks | 417 (2,762) | 137 (26,341) | -81.4% |
| MC4 | 40% | 4 weeks | 136 (1,377) | 71 (9,237) | -90.3% |
| SM7 | 80% | 6 weeks | 160 (2,423) | 89 (9,250) | -87.9% |
| SM8 | 80% | 8 weeks | 214 (8,495) | 104 (14,308) | -85.9% |
